# Development of a dictionary of information items inspired by common data models to support health research data access requests

**DOI:** 10.23889/ijpds.v9i5.2502

**Published:** 2024-09-18

**Authors:** Carrie-Anne Whyte, Carmen La, Ali Anis, David Yang, Jean-François Ethier, Mark McGilchrist

**Affiliations:** 1 Canadian Institute for Health Information; 2 Population Data British Columbia; 3 Université de Sherbrooke; 4 University of Dundee

A network of organizations collaborates to provide services to facilitate multi-regional research across Canada. To streamline the data access process, the network is developing a dictionary of information items (InfoItems), providing a label and definition that represent a semantic sourced from an ontology (e.g., “human birth date” from a demography ontology). This allows for a standardized and shareable means to express data requirements.

Inspired by health-research common data models (CDMs) like those from the Observational Medical Outcomes Partnership (OMOP) and the Canadian Network for Observational Drug Effects (CNODES), approximately 60 InfoItems were developed by the network. As a pilot exercise, four data centres mapped their data assets to the InfoItems to ensure they are relevant to the Canadian health data context. This has laid the foundation for InfoItems as a basic data harmonization mechanism.

The new InfoItems-based data dictionary promotes the standardization of data asset contents across the network’s data centres. Researchers can use the dictionary to specify a project’s data requirements, establishing details like data availability across multiple regions, for inclusion in their data access request.

The InfoItems dictionary is pivotal in simplifying data access requests, promoting multi-regional research in Canada. This resource can simultaneously safeguard the quality of analytical results and optimize data extraction into existing data models (e.g., health-research CDMs). Ongoing improvements in this area aim to enhance user experience and facilitate quality research.

